# Enhanced oxidative stress resilience in *C. elegans acox-1.1* mutants through CTL-3 and proteasomal regulation

**DOI:** 10.3389/ebm.2026.10796

**Published:** 2026-03-13

**Authors:** Woori Bae, Mina Norman, Myon Hee Lee

**Affiliations:** 1 Department of Biochemistry and Molecular Pharmacology, New York University School of Medicine, New York, NY, United States; 2 Department of Biochemistry, College of Life Science and Biotechnology, Yonsei University, Seoul, Republic of Korea; 3 Department of Internal Medicine, Hematology/Oncology Division, Brody School of Medicine at East Carolina University, Greenville, NC, United States

**Keywords:** *C. elegans* aging, catalase (CTL-3), oxidative stress adaptation, peroxisomal β-oxidation, proteasome dysfunction

## Abstract

Oxidative stress is a primary driver of aging, necessitating robust cellular adaptation mechanisms. While peroxisomal β-oxidation and proteasomal degradation are known to influence stress responses, their functional crosstalk remains elusive. In this study, we show that *C. elegans acox-1.1* mutants, despite having a shortened lifespan under normal conditions, exhibit a paradoxical resistance to mild chronic oxidative stress (1 mM paraquat, PQ) compared to wild-type worms. This PQ-induced resistance in *acox-1.1* mutants was independent of the canonical SKN-1 pathway but required the peroxisomal catalase CTL-3. RNA-mediated knockdown of *ctl-3* largely abolished the stress resistance of *acox-1.1* mutants, leading to rapid mortality. Proteomic and biochemical analyses revealed that *acox-1.1* mutants possess reduced levels of PAS-5, a core 20S proteasome subunit, resulting in impaired proteasomal assembly and accumulation of ubiquitinated (Ub) substrates under basal conditions. Intriguingly, exposure to 1 mM PQ significantly reduced the Ub-smear in *acox-1.1* mutants, suggesting a metabolic shift where the cell prioritizes ROS scavenging over ATP-dependent protein degradation. Under oxidative stress, *acox-1.1* mutants bypass defective proteasomal machinery and redirect energy toward CTL-3-mediated antioxidant defense. This study identified a peroxisomal adaptation mechanism whereby reduced proteasome complexity, coupled with enhanced ROS-regulatory machinery, confers survival advantages under specific oxidative challenges.

## Impact statement

Our study demonstrates that *C*. *elegans acox-1.1* mutants adapt to peroxisomal β-oxidation deficiency by activating CTL-3-mediated antioxidant defenses and proteasome regulation, resulting in increased stress resilience under mild oxidant stress. These findings uncover a mechanistic link between metabolic dysfunction, ROS detoxification, and proteostasis, which offer new perspectives on cellular adaptation and organismal resilience.

## Introduction

Peroxisomes are vital organelles responsible for lipid metabolism, detoxification, and redox homeostasis. A key function of peroxisomes is β-oxidation of very long-chain fatty acids (VLCFAs), a process that differs from mitochondrial β-oxidation by producing hydrogen peroxide (H_2_O_2_) instead of ATP [1, 2]. While H_2_O_2_ has long been considered a metabolic byproduct, emerging evidence suggests that it plays an integral role in redox signaling and cellular stress adaptation [3].

In *Caenorhabditis elegans* (*C. elegans*), acyl-CoA oxidase 1 (*acox-1*) catalyzes the initial step of peroxisomal β-oxidation, converting acyl-CoA into 2-trans-enoyl-CoA [4]. Loss of *acox-1.1* results in metabolic dysregulation, characterized by lipid accumulation and increased oxidative stress, which may activate compensatory stress response pathways [5]. The disruption of peroxisomal VLCFA metabolism has been closely linked to oxidative stress and cellular dysfunction, as defective peroxisomal activity leads to reactive oxygen species (ROS) accumulation and subsequent macromolecular damage [6, 7].

Oxidative stress exerts a profound influence on protein homeostasis (proteostasis) by altering protein conformation and increasing the accumulation of oxidized, dysfunctional proteins [8]. To counteract this, cells employ the ubiquitin-proteasome system (UPS) and the immunoproteasome, which degrade oxidatively damaged proteins and maintain proteostasis [9]. Notably, oxidative stress has been demonstrated to modulate proteasome activity, highlighting its central role in cellular stress responses [10, 11]. The proteasome serves as a critical regulator of proteostasis, rapidly degrading oxidized and misfolded proteins under oxidative stress conditions [12]. Among its components, the 20S proteasome functions independently of ubiquitination, enabling a rapid and selective response to ROS-induced proteotoxic stress [13, 14]. Declining proteasome activity has been directly associated with aging and age-related pathologies, underscoring its essential role in longevity [15].

PAS-5, a regulatory subunit of the 20S proteasome, is indispensable for proteasome assembly and function [14, 16]. In *C. elegans*, *pas-5* depletion via RNAi phenocopies the loss of *pas-4*, further emphasizing its role in proteostasis [11]. Previous work in an *xpa-1* mutant background, using transcriptomic and proteomic profiling, revealed that *pas-5* depletion triggered upregulation of other proteasome components, particularly *pbs-4,* in an SKN-1-dependent manner, indicating activation of oxidative stress responses when DNA repair is compromised [17].

A key regulator of oxidative stress resistance in *C. elegans* is SKN-1, the ortholog of mammalian Nrf2, which modulates antioxidant defense, detoxification, and metabolic homeostasis [18, 19]. Under acute oxidative stress, SKN-1 activation drives expression of canonical detoxification genes, such as *gcs-1* and *gst-4*, enabling rapid clearance of ROS [18]. Under persistent or chronic stress conditions, previous studies have shown that SKN-1-dependent transcriptional outputs can be redirected away from classical detoxification genes toward pathways involved in metabolic adaptation and stress accommodation [20]. Consistent with this concept, chronic exposure to low-dose paraquat (0.25–1 mM) induces adaptive stress responses [21] and longevity-associated signaling in *C. elegans*, highlighting a mode of oxidative stress distinct from acute toxicity [22].

Recent advancements in optogenetic control of ROS production have further underscored the importance of precisely regulated ROS dynamics in metabolic adaptation and stress resilience [23]. The growing body of evidence supporting oxidative stress as a key determinant of lifespan regulation in *C. elegans* further strengthens the significance of these pathways in aging [24]. While oxidative stress, proteostasis, and peroxisomal metabolism are well-established players in aging, the role of the proteasome in integrating these pathways remains underexplored. Since PAS-5 is crucial for proteasome stability and function, its regulation under oxidative stress may uncover novel adaptive mechanisms contributing to lifespan extension. Recent findings suggest that peroxisomal dysfunction triggers compensatory stress responses involving proteasomal adaptation [25, 26]. However, whether PAS-5 directly contributes to oxidative stress resistance in *acox-1.1(ok2257)* mutants remains unknown.

In this study, we investigated the functional significance of *acox-1.1* in oxidative stress adaptation and aging, specifically in the context of peroxisomal β-oxidation deficiency. We examined (1) the impact of *acox-1.1* deficiency on ROS accumulation and oxidative stress resistance, (2) the reduced expression of the proteasome subunit PAS-5 and its impact on the accumulation of ubiquitinated substrates in *acox-1.1(ok2257)* mutants, the (3) *acox-1.1(ok2257)* mutants prioritize antioxidant defense over proteasomal activity under severe oxidative challenges, as evidenced by the reduction of Ub-conjugates. By elucidating the molecular crosstalk between peroxisomal dysfunction, oxidative stress, and proteostasis, our findings provide mechanistic insights into how metabolic defects can trigger adaptive stress responses that promote resilience against environmental stress.

## Materials and methods

### Maintenance of *C. elegans* strains

The *C. elegans* strains used in this study were N2 Bristol (wild-type) and *acox-1.1(ok2257)* mutant worms. All worms were cultured as previously described [27].

### RNA interference (RNAi) experiments

RNAi was performed using the feeding method. The *ctl-3*(RNA*i*) clone was constructed by amplifying a specific genomic region (approximately 800 bp) of the *ctl-3* gene from *C. elegans* genomic DNA using the following primers containing *Xba*I and *Hind*III restriction sites at their 5′ ends: forward, 5′-ACT​TCT​AGA​CCG​CCG​TGC​TCA​CCG​CC-3′ and reverse, 5′-TCT​AAG​CTT​GTT​CGA​ATG​TCA​TCA​CTT​G-3’. The resulting PCR product was digested with *Xba*I and *Hind*III and ligated into the L4440 vector at the corresponding restriction sites. The resulting construct was then transformed into *E. coli* HT115 (DE3) RNAi bacteria. For feeding RNAi experiments, *ctl-3* RNAi bacteria clones were cultured overnight in LB medium at 37 °C. The cultured bacteria were then seeded onto NGM agar plates containing 1 mM IPTG and 100 μg/mL ampicillin to induce double-stranded RNA expression.

### Quantitative real-time polymerase chain reaction (qRT-PCR)

Total RNA was extracted from age-synchronized worms using TRIzol Reagent (Invitrogen, Carlsbad, CA, USA; Thermo Fisher Scientific, Waltham, MA, USA) and purified with the RNeasy Kit (QIAGEN, Hilden, Germany). Reverse transcription was performed using the Transcriptor First Strand cDNA Synthesis Kit (Roche, Basel, Switzerland) with oligo(dT) primers. Quantitative real-time PCR (qRT-PCR) was conducted using iQ SYBR Green Supermix (Bio-Rad Laboratories, Hercules, CA, USA) according to the manufacturer’s instructions. Amplification products were analyzed on a CFX Connect™ Real-Time PCR Detection System (Bio-Rad Laboratories, Hercules, CA, USA). Relative mRNA expression levels were calculated using the ΔΔCT method, with *ama-1* or *act-1* serving as reference genes for normalization. It minimizes potential errors caused by subtle fluctuations in a single housekeeping gene, thereby ensuring a more reliable normalization base. All biological replicates within each experiment were consistently normalized using *ama-1* as the reference gene. The primers used for qRT-PCR in this study are listed in Supplementary Table S1.

### Endogenous ROS measurement

Measurement of ROS was performed as described [28]. Worms were transferred to 100 µL PBS-T (0.1% Tween 20) using L4-stage worms. The worms were frozen in liquid nitrogen and then sonicated for 20 s and 90 s, a process repeated five times. All samples were analyzed with five technical replicates in 96-well black-bottom, clear plates. Samples were incubated with 50 µM DCF-DA at 37 °C. Fluorescence was measured using the Victor 5 multilabel plate reader (Perkin Elmer, USA) with excitation at 485 nm and emission at 640 nm for 2 h, with readings taken every 10 min. The experiments were normalized to PBS-only samples, with five technical replicates for each condition.

### Paraquat (PQ)-induced oxidative stress survival assay

L4-stage wild-type and *acox-1.1(ok2257)* worms were exposed to 1 mM PQ and basal conditions, with Day 0 corresponding to the L4 stage. For each experiment, 36 to 60 worms were used per biological repeat. All assays were performed with 3 or 4 independent replicates at 20 °C. Statistical analyses of lifespan curves were performed using the log-rank (Mantel-Cox) test in Prism 10. Hazard ratios were calculated with the Cox proportional-hazard model.

### Western blotting

Wild-type and transgenic worms were harvested following PQ treatment and lysed in Laemmli sample buffer (Bio-Rad Laboratories, Inc., Hercules, CA, USA) by boiling for 10 min. Equal amounts of protein were separated by 10% SDS-PAGE (Bio-Rad Laboratories, Inc., Hercules, CA, USA). Protein-transferred membranes were blocked with 5% (w/v) non-fat milk (Biotium, Inc., Fremont, CA, USA) in Tris-buffered saline (TBS) containing 0.1% Tween 20 (TBST) for 30 min at 25 °C. Primary antibodies (anti-total Ub, Santa Cruz 1:200, anti-Tub, 1:1000, DSHB AA4.3) were incubated overnight at 4 °C in 5% non-fat dry milk in TBST, followed by incubation with secondary antibodies (HRP-goat anti-mouse 1:5000, Invitrogen 31430) for 2 h at 25 °C. Western blot images were obtained using the LI-COR C-DiGit Chemiluminescence Western Blot Scanner and Image Studio software (LI-COR, Lincoln, Nebraska, USA).

### Sample preparation for two-dimensional electrophoresis (2DE)

We performed as previously described [29]. Worms were washed with distilled water and resuspended in sample buffer A (50 mM Tris, 5 mM EDTA, 7 M urea, 2 M thiourea, 4% CHAPS, and protease inhibitor). The suspensions were sonicated on ice for 30 s, and the soluble fractions were obtained by centrifugation at 36,000 × *g* for 40 min at 4 °C. Protein concentrations were determined using the Bradford method. Aliquots were stored at −70 °C until use. Protein samples (100 µg for analytical gels and 1 mg for preparative gels) were diluted in sample buffer B (7 M urea, 2 M thiourea, 2% v/v IPG buffer, pH 3–10 nonlinear (Amersham Biosciences), 2% CHAPS, 15 mM dithiothreitol, and a trace of bromophenol blue). The protein mixtures were applied onto IPG strips (Immobiline DryStrip, pH 3–10 nonlinear, 18 cm; Amersham Biosciences) prehydrated with the protein solution at 20 °C for 14 h. Isoelectric focusing (IEF) was performed at 20 °C with a current limit of 50 mA/strip using the following protocol [29]. After focusing, IPG strips were equilibrated for 20 min with gentle agitation in buffer containing 375 mM Tris-HCl (pH 8.8), 6 M urea, 2% SDS, 5 mM tributyl phosphine, 2.5% acrylamide solution, and 20% glycerol. For the second dimension, vertical SDS-PAGE was performed using 9–16% gradient slab gels (180 × 200 × 1.5 mm). Equilibrated strips were trimmed and sealed onto the gels with 0.5% agarose in running buffer (24.8 mM Tris (pH 8.3), 192 mM glycine, 0.1% SDS, and a trace of bromophenol blue). Electrophoresis was performed at a constant current of 15 mA per gel. Gels were fixed in 40% methanol and 5% phosphoric acid for at least 1 h and stained overnight with Coomassie Brilliant Blue G-250, destained, scanned using a GS-710 image scanner (Bio-Rad), and analyzed with Melanie 3 software (GeneBio).

### MALDI-TOF mass spectrometry analysis

We performed as previously described [29]. Protein spots were excised from the gels using pipette tips with the ends removed to accommodate varying diameters. Excised gel pieces that were transferred to microtubes were destained and dehydrated in 50 µL acetonitrile for 5 min at room temperature. The dried gels were rehydrated on ice for 45 min with 10 µL of trypsin solution (10 mg/mL in 25 mM ammonium bicarbonate, pH 8.0). Excess solution was removed, and proteins were digested at 37 °C for 24 h. The resulting peptides were extracted with POROS R2 beads [30] and analyzed using a Voyager DE Pro MALDI-TOF mass spectrometer (Applied Biosystems, Foster City, CA, USA). For sample preparation, 0.5 µL of α-cyano-4-hydroxycinnamic acid matrix solution was mixed with the peptide solution. Time-of-flight (TOF) measurements were performed under the following conditions: accelerating voltage of 20 kV, grid voltage of 75%, guide wire voltage of 0%, a delay time of 120 ns, and a low-mass gate of 500 Da. Internal calibration was performed using the autodigestion peaks of porcine trypsin (M + H^+^, m/z 842.5090 and 2211.1064). Peptide mass profiles were searched against the National Center for Biotechnology Information (NCBI) database using MS-Fit 3.2 (University of California, San Francisco;^1^) and ProFound (version 4.7.0; The Rockefeller University;^2^), applying a mass tolerance of 20 ppm for measurements acquired in reflector mode.

## Results

### *acox-1.1(ok2257)* mutants exhibit enhanced oxidative stress resistance under mild chronic oxidative stress

**FIGURE 1:**
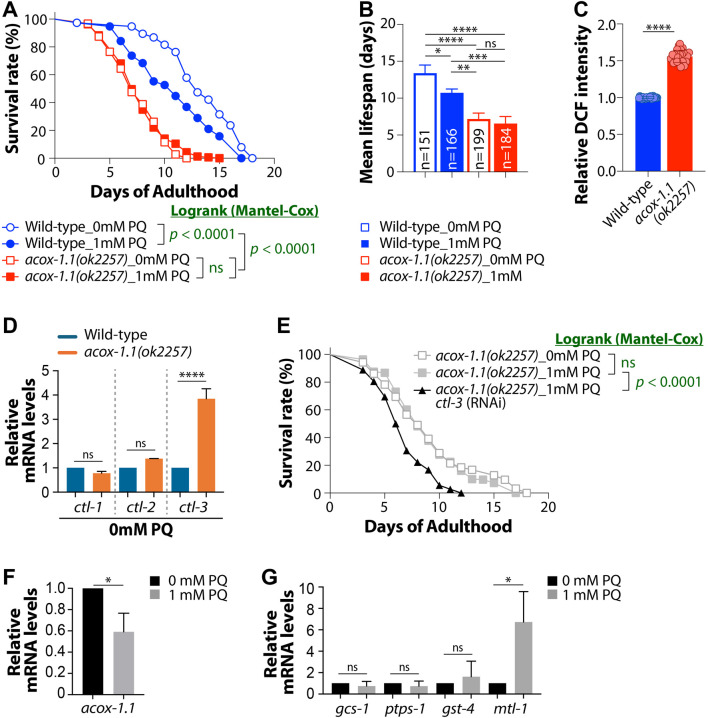
Characterization of a CTL-3-dependent oxidative stress adaptive response in *acox-1.1(ok2257)* mutants. **(A)** Survival analysis under basal and PQ-induced oxidative stress conditions demonstrates that *acox-1.1(ok2257)* mutants exhibit increased resistance to PQ-induced mortality compared to wild-type worms, indicating enhanced oxidative stress tolerance. **(B)** Mean lifespan. **(C)** Measurement of intracellular ROS levels reveals a significant increase in *acox-1.1(ok2257)* mutants relative to wild-type worms, suggesting elevated oxidative stress due to *acox-1.1* deficiency. **(D)** Quantitative RT-PCR analysis of catalase gene expression shows that *ctl-3*, but not *ctl-1* or *ctl-2*, is selectively upregulated in *acox-1(ok2257)* mutants, indicating specific activation of a CTL-3-mediated ROS scavenging pathway. Data represent means ± SD from two biological replicates. **(E)** CTL-3 is required for oxidative stress resistance in *acox-1.1(ok2257)* mutants. RNA*i*-mediated knockdown of *ctl-3* abolishes the enhanced survival of *acox-1.1(ok2257)* mutants under 1 mM PQ treatment. **(F)** Quantification of *acox-1.1* mRNA levels shows a marked reduction under mild chronic oxidative stress conditions (1 mM PQ), reflecting the impaired peroxisomal β-oxidation cycle. **(G)** Expression analysis of oxidative stress-responsive genes (*gcs-1*, *ptps-1*, *gst-4*, and *mtl-1*) following 1 mM PQ treatment indicates a significant upregulation of *mtl-1* in *acox-1.1(ok2257)* mutants compared to wild-type worms. Data represent means ± SD from three biological replicates.

To examine the effects of *acox-1.1* deficiency on lifespan under oxidative stress, we compared the survival of wild-type and *acox-1.1(ok2257)* mutant worms under basal conditions and in the presence of 1 mM paraquat (PQ). Under normal growth conditions, *acox-1.1(ok2257)* mutants exhibited a significantly shorter mean lifespan than wild-type worms (Figures 1A,B), consistent with impaired metabolic homeostasis. Upon PQ treatment, wild-type worms showed a significant reduction in mean lifespan relative to untreated conditions (Figures 1A,B). In contrast, *acox-1.1(ok2257)* mutants did not exhibit PQ-induced lifespan reduction and instead maintained a comparable or slightly increased mean lifespan under oxidative stress (Figures 1A,B). Consistently, Log-rank (Mantel-Cox) analysis revealed that wild-type worms showed a significant difference in survival distributions with and without PQ treatment (*p* < 0.001), whereas *acox-1.1(ok2257)* mutants exhibited no significant difference under the same conditions (Figure 1A). Notably, *acox-1.1(ok2257)* mutants maintained high viability during early and mid-adult stages under PQ treatment, with a delayed onset of mortality compared to wild-type worms. These results indicate that loss of *acox-1.1* confers enhanced resistance to oxidative stress, potentially through metabolic adaptations or activation of stress-response pathways. This interpretation is consistent with previous studies linking peroxisomal function to lifespan regulation in *C. elegans* [31] and supports the notion that metabolic rewiring in response to oxidative stress contributes to stress resistance and survival outcomes [31, 32].

### Elevated endogenous H_2_O_2_ levels in *acox-1.1(ok2257)* mutants

Since *acox-1.1* plays a role in peroxisomal β-oxidation, we next investigated whether its loss alters ROS homeostasis. Intracellular hydrogen peroxide (H_2_O_2_) accumulation was measured using DCF fluorescence intensity over a 2-h period (Figure 1C; Supplementary Figure S1). The results showed that *acox-1.1(ok2257)* mutants consistently exhibited higher DCF fluorescence intensity than wild-type controls at all time points, indicating a sustained increase in endogenous ROS levels. At 120 min, *acox-1.1(ok2257)* mutants displayed a marked elevation in fluorescence signal, suggesting a significant accumulation of ROS (*p* < 0.001) (Supplementary Figure S1). The continuous rise in fluorescence indicates that the loss of *acox-1.1* disrupts peroxisomal function, leading to impaired H_2_O_2_ detoxification and an oxidative stress-prone cellular state. In previous studies, *acox-1.1* (RNAi) did not significantly alter H_2_O_2_ levels compared with wild-type worms [31], whereas *acox-1.1(ok2257)* mutants accumulate internal ROS. Despite increased ROS levels, the prolonged survival of *acox-1.1(ok2257)* mutants under oxidative stress conditions implies the presence of adaptive mechanisms that mitigate ROS-induced damage, possibly through enhanced antioxidant defense or stress tolerance pathways.

### Differential expression of *ctl* genes in *acox-1(ok2257)* mutants

Given that the *acox-1.1(ok2257)* mutants lead to elevated endogenous levels of ROS, we examined the expression of *C. elegans* catalase genes involved in ROS detoxification to assess potential compensatory responses. In *C. elegans*, *ctl-1* encodes a cytosolic catalase, whereas *ctl-2* and *ctl-3* encode peroxisomal catalases, highlighting distinct subcellular localization among the family members [33]. Elevated catalase activity can mitigate tissue metabolic damage by reducing toxic H_2_O_2_ levels [34]. qRT-PCR analysis revealed that the expression of *ctl-1* and *ctl-2* genes remained unchanged (*p* > 0.05) compared to wild-type worms, whereas *ctl-3* was significantly upregulated (*p* = 0.0006) (Figure 1D). These results suggest that loss of *acox-1.1* alters the expression of ROS scavenging genes, with *ctl-3* exhibiting the most pronounced induction, potentially compensating for increased ROS levels. Peroxisomes are increasingly recognized as key organelles in cellular adaptation to oxidative stress, given their capacity to both generate and detoxify ROS. Both *ctl-1* and *ctl-2* have been implicated in lifespan regulation [35], while the role of *ctl-3* remains uncharacterized. A recent study demonstrated that peroxisomes actively participate in the cell-wide response to transient heat shock, revealing a critical role for organelle communication in coordinating organismal stress responses [36]. Under such extreme oxidative environments, the cell appears to shift its energetic priorities toward a survival-centric mode, prioritizing the clearance of oxidized proteins via specific antioxidant defenses such as CTL-3. We observed that the oxidative stress resistance of *acox-1.1(ok2257)* mutants is heavily dependent on this axis; indeed, *ctl-3* RNA*i* completely abolishes this resistance and leads to a short lifespan (Figure 1E). This suggests that, in the face of severe oxidative stress, the failure of the Ub-proteasome system (UPS) complex assembly is superseded by a CTL-3-mediated stress clearance pathway to maintain cellular proteostasis and survival.

### Transcriptional response to oxidative stress: acox-1.1 downregulation and oxidative stress gene mtl-1 upregulation

To assess the effect of oxidative stress on *acox-1.1* transcription, we quantified *acox-1.1* mRNA levels using qRT-PCR in wild-type worms with and without PQ treatment. As shown in Figure 1F, *acox-1.1* expression was significantly downregulated in PQ-treated wild-type worms compared to untreated controls (*p* = 0.034). This result suggests that oxidative stress negatively regulates *acox-1.1* transcription, possibly as part of a broader metabolic adaptation to ROS accumulation. We also analyzed the mRNA levels of *gcs-1, ptps-1, gst-4,* and *mtl-1* to determine whether oxidative stress modulates the expression of key antioxidant defense genes (Figure 1G). Consistent with previous reports, SKN-1 target detoxification genes were suppressed under mild chronic oxidative stress, likely conserving energy to support adaptive responses rather than acute detoxification [20, 37]. Notably, only one of the *daf-16* downstream genes, *mtl-1,* was significantly upregulated following PQ treatment. This finding suggests that *acox-1.1(ok2257)* mutants may adapt through preferential activation of DAF-16 rather than SKN-1 under mild chronic oxidative stress. Together, these findings indicate that oxidative stress triggers transcriptional alterations that downregulate *acox-1.1* expression and induce a distinct ROS-response pathway from the canonical SKN-1-mediated oxidative stress defense, thereby enhancing stress resilience.

### Proteomic analysis reveals reduced PAS-5 levels and oxidative stress-mediated suppression of ubiquitination in *acox-1.1(ok2257)* mutants

**FIGURE 2:**
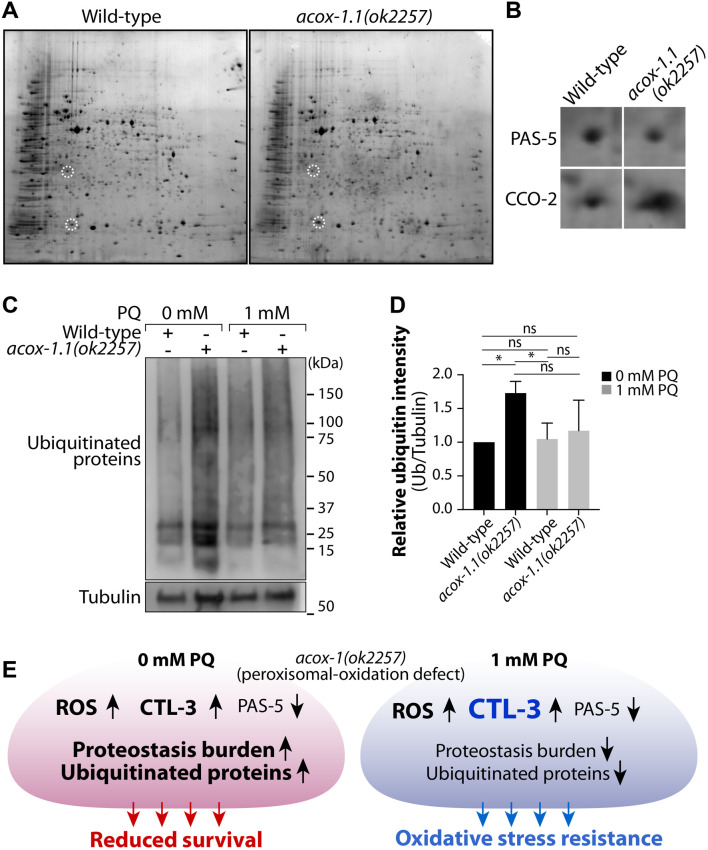
Proteostasis alterations and a proposed adaptive model in *acox-1.1(ok2257)* mutants. **(A)** Representative 2D electrophoresis gel images comparing protein extracts from mixed-stage wild-type and *acox-1.1(ok2257)* mutant worms. **(B)** Enlarged views of differentially expressed protein spots are indicated, with PAS-5 showing a decrease and CCO-2 showing a notable increase in *acox-1.1(ok2257)*. **(C)** Western blot analysis of ubiquitinated proteins (Ub) in wild-type and *acox-1.1(ok2257)* mutants under basal conditions and following oxidative stress conditions (1 mM PQ). Upon PQ treatment, ubiquitin-conjugated protein levels are markedly reduced *in acox-1.1(ok2257)* mutants, indicating an oxidative stress-dependent alteration in ubiquitination dynamics. (The original Western blot images are included in Supplementary Figure S2). **(D)** Relative ubiquitin intensity. Quantification was performed using ImageJ software. **(E)** Proposed model illustrating an adaptive response to oxidative stress in *acox-1.1(ok2257)* mutants, highlighting the interplay between elevated ROS, reduced proteasome activity via PAS-5 downregulation, and elevated ROS-mediated regulatory machineries (upregulation of CTL-3). Reduced PAS-5 levels may impair proteasome integrity, thereby contributing to an adaptive oxidative stress-resistant state.

To identify proteins differentially expressed in *acox-1.1(ok2257)* mutants that may contribute to their oxidative stress-resistant lifespan extension, we performed two-dimensional (2D) gel electrophoresis to compare global protein expression profiles with wild-type worms (Figure 2A). Several protein spots displayed altered intensity in *acox-1.1(ok2257)* mutants, indicating widespread changes in protein abundance. Among the significantly altered proteins, PAS-5, a key regulatory subunit of the 20S proteasome, exhibited an approximately one-third reduction in *acox-1.1(ok2257)* mutants compared to wild-type worms. PAS-5 was detected in the wild-type proteome (white circle, left panel), whereas its levels were markedly diminished in *acox-1.1(ok2257)* mutants (right panel). In addition, CCO-2 (cytochrome c oxidase subunit 2, COX2) showed a notable increase in *acox-1.1(ok2257)* mutants. These changes were further validated by a zoomed-in analysis (Figure 2B). Given the essential role of PAS-5 in proteasome assembly and function, these findings suggest that peroxisomal dysfunction may indirectly impact proteostasis by modulating proteasomal activity, possibly through metabolic stress-induced alterations in protein turnover. Moreover, *acox-1.1(ok2257)* mutants manage ROS through SKN-1-independent pathways, potentially involving DAF-16, with increased mitochondrial CCO-2, supporting oxidative stress adaptation. Under basal conditions, reduced PAS-5 levels in *acox-1.1(ok2257)* mutants are associated with impaired proteostasis, as reflected by the accumulation of ubiquitinated proteins, consistent with incomplete assembly or reduced activity of the 20S proteasome core. Surprisingly, upon exposure to low-dose oxidative stress, this phenotype is reversed, with a marked reduction in ubiquitin-conjugated protein smearing (Figures 2C,D). We suggested that this decrease in ubiquitinated substrate under oxidative stress does not reflect restored proteasome activity, but rather inhibition of the ubiquitination machinery itself. Elevated reactive oxygen species (ROS) are known to oxidize critical cysteine residues within the catalytic sites of E1, E2, and E3 enzymes, thereby impairing ubiquitin activation and conjugation steps [38, 39]. Together, these findings suggested that PAS-5 downregulation and oxidative stress-mediated suppression of ubiquitination converge to reshape proteostasis in *acox-1.1(ok2257)* mutants.

## Discussion

Based on our findings, we propose a mechanistic model (Figure 2E) in which loss of *acox-1.1* disrupts VLCFA metabolism, leading to oxidative stress and endogenous ROS accumulation. The resulting oxidative stress activates compensatory detoxification mechanisms, including the induction of catalase, which neutralizes ROS, and modulation of the proteasome component PAS-5, potentially contributing to proteostasis regulation. Given that PAS-5 is a regulatory subunit of the 20S proteasome, the observed reduction in PAS-5 levels in *acox-1.1(ok2257)* mutants raises the question of whether proteasome activity is compromised under oxidative stress conditions. Future studies will be required to assess proteasome subunit composition and catalytic function in *acox-1.1(ok2257)* mutants. Ultimately, these findings suggest that oxidative stress establishes an adaptive threshold in *acox-1.1(ok2257)* mutants, allowing them to survive prolonged exposure to ROS by engaging antioxidant defenses and proteostasis mechanisms. Consistent with this model, RNAi-mediated depletion of *ctl-3* abolishes the oxidative resilience of *acox-1.1(ok2257)* mutants, indicating that catalase-dependent antioxidant defense is essential for their adaptive survival under oxidative stress. Further investigations, including genetic epistasis analyses and proteasome activity assays, will be necessary to fully elucidate the regulatory interplay between metabolic stress, proteasome function, and oxidative stress adaptation.

## Data Availability

The raw data has been deposited in Figshare (DOI: 10.6084/m9.figshare.31545961).
