# Supplementary material for: Enhanced oxidative stress resilience in C. elegans acox-1.1 mutants through CTL-3 and proteasomal regulation
Source: Exp Biol Med (Maywood). 2026 Mar 13;251:10796. doi: 10.3389/ebm.2026.10796 (PMC13021563; doi:10.3389/ebm.2026.10796)
Supplement: Supplementary file 1 [file Supplementaryfile1.docx]

**Supplementary Table 1. Primer information**

| **Primer name** | **Sequence (5’ to 3’)** |
| --- | --- |
| *acox-1.1(F)* | ACTCATGCGATGGCTGCACAG |
| *acox-1.1(B)* | CGCTCGAATGAGCCACTTGGCT |
| *act-1(F)* | AAGTCCTACGAACTTCCTGACG |
| *act-1(B)* | GAGATCCACATCTGTTGGAAGG |
| *ama-1(F)* | CCTACGATGTATCGAGGCAAA |
| *ama-1(B)* | CCTCCCTCCGGTGTAATAATG |
| *ctl-1(F)* | CACTGATCGAGGTCGGCAAGATG |
| *ctl-1(B)* | GATGGTAATGCGTGTCCGTGTAGG |
| *ctl-2(F)* | GACAATCAGCAACATGCTCC |
| *ctl-2(B)* | CTGGCACATTCTCTCCCGAG |
| *ctl-3(F)* | GTGGTCCAATGCTAATGCAGGACA |
| *ctl-3(B)* | AGTATCCATGAGCACCACCACCTT |
| *gcs-1(F)* | AATCGATTCCTTTGGAGACC |
| *gcs-1(B)* | ATGTTTGCCTCGACAATGTT |
| *ptps-1(F)* | TGGTGTATGACCTGGCAAAG |
| *ptps-1(B)* | CGGATTTCAGCTTCTCGAAC |
| *gst-4(F)* | TGCTCAATGTGCCTTACGAG |
| *gst-4(B)* | AGTTTTTCCAGCGAGTCCAA |
| *mtl-1(F)* | AGTGCGGAGACAAATGTGAATGC |
| *mtl-1(B)* | AGCAGTTCCCTGGTGTTGATGG |

**Supplementary Figures**

**
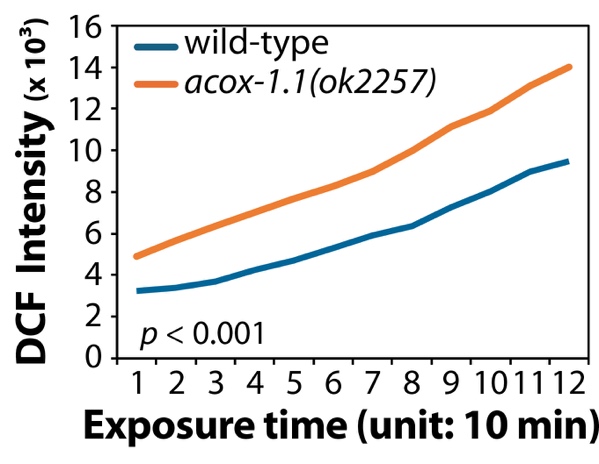
**

**Supplementary Figure S1. Measurement of intracellular ROS in worms using 2',7'-dichlorofluorescein diacetate (H_2_DCFDA), a cell permeant tracer.** ROS levels were significantly increased in *acox-1(ok2257)* mutants relative to wild-type worms, suggesting elevated oxidative stress due to *acox-1.1* deficiency.

**
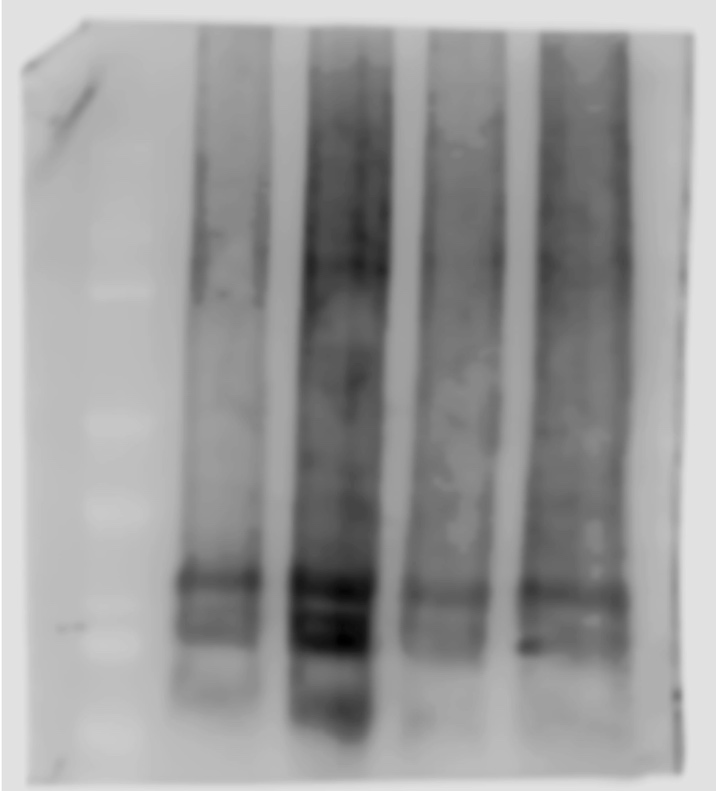

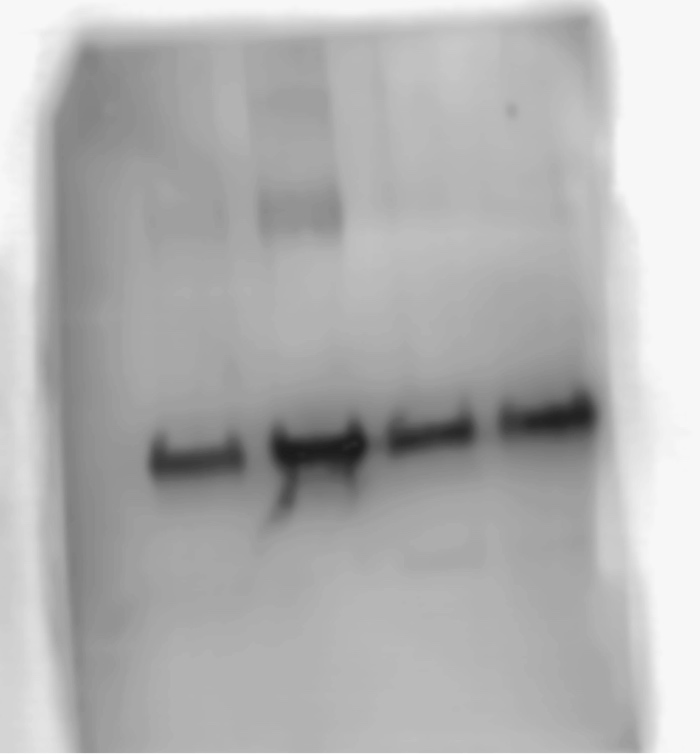
**

**Supplementary Figure S2. Original western blot images for Figure 2C.** Ubiquitinated proteins (left) and tubulin (right).
